# Supplementary material for: A Single-Cell Atlas of an Early Mongolian Sheep Embryo
Source: Vet Sci. 2023 Aug 28;10(9):543. doi: 10.3390/vetsci10090543 (PMC10536297; doi:10.3390/vetsci10090543)
Supplement: Supplementary file 1 [file vetsci-10-00543-s001.zip › vetsci-2450971-supplementary.pdf]

Supplementary file for: The mongolia sheep early embryo transcriptional cell atlas

The file includes:
Table S1-S3
Supplementary Figures S1-S5

Table S1: The number of different cell types in Ujumqin sheep E16 embryos

Cell Type	Cell Counts	Mean Gene Counts
Blood progenitor	617	1208.627229
Cardiomyocytes	37	586.8378378
Caudal neuroectoderm	2524	285.3716323
Endothelium	338	501.352071
Erythroid	4975	1608.089045
ExE endoderm	258	1020.581395
Gut	431	1215.213457
Haematoendothelial progenitors	158	3235.987342
Mesenchyme	893	607.0414334
Notochord	391	2200.217391
Paraxial mesoderm	144	2470.256944
Somite	1989	3094.518351
Spinal cord	1801	583.6435314

TableS2: The number of different cell types in Hulunbuir sheep E16 embryos

Cell Type	Cell Counts	Mean Gene Counts
Endothelium	235	745.4553191
Gut	391	2121.815857
Haematoendothelial progenitors	487	2796.149897
Notochord	544	2113.382353
Paraxial mesoderm	1118	2104.246869
Primitive streak/Cardiomyocytes	1505	817.7860465
Somite	5098	2491.547666
Spinal cord	205	1873.419512

TableS3: Primers used in the study.

Gene	Forward Primer (5'-3')	Reverse Primer (5'-3')	Organism
HOXB9	CGAAGGAAGCGAGGACAAAGAGAG	CAGCGTCTGGTATTTGGTGTAGGG	sheep
ACLY	ATCACCGAGGTCTTCAAGGAGGAG	GCTGTCACCATCAGGCACATCTC	sheep
FASN	AACGCTGTGGTGCTGGAGATTG	GTTGTCCCTGTGGTCCTTCTTCATC	sheep
HOXC12	GGGCGAGCATAATCTCCTGAATCC	GCGGAAGTTGGGAAAGTAGAAGGTG	sheep
AHSG	CCAGGTTCTGTCTCTGTGGAGTTTG	AGGTTGCACTTGGTTGGATCTACG	sheep
AFP	GCTTGGTGGTGGATGAGACATACG	TGGCTTCTGCTTCACGAGGTTAATG	sheep
FST	TCAAGTGGATGATTTTCAACGG	GTTCTTCTTGTTCATTCGGCAT	sheep
VIM	TCAATGACCGCTTCGCCAACTAC	GCTCTCGCATCTCCTCCTCGTAG	sheep
ACTB	TCCTGCGGCATTCACGAAACTAC	GTGTTGGCGTAGAGGTCCTTGC	sheep
MYC	TCAACGTCAGCTTCGCCAACAG	AAGTTCTCCTCCTCGTCGCAGTAG	sheep
YAP	AGAGGCCTGGCGCATCAAAA	CCAACAAGGTTCCAGCCCAT	sheep
TAZ	TGCGGGCAGAGAACAAGTCA	CTGGCTTGCAGGTGGTTGTG	sheep

Supplementary Figure

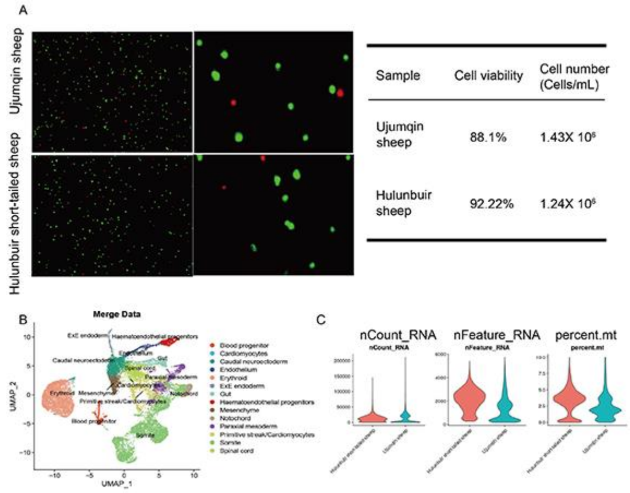

Fig. S1 Cell viability detection, integrate analysis and quality control analysis in Ujumqin and Hulunbuir sheep.

(A) Single cell viability and concentration detection in Ujumqin and Hulunbuir sheep. **(B)** Integrating Ujumqin and Hulunbuir sheep analysis using UMAP. **(C)** Quality control analysis in Ujumqin and Hulunuir sheep.

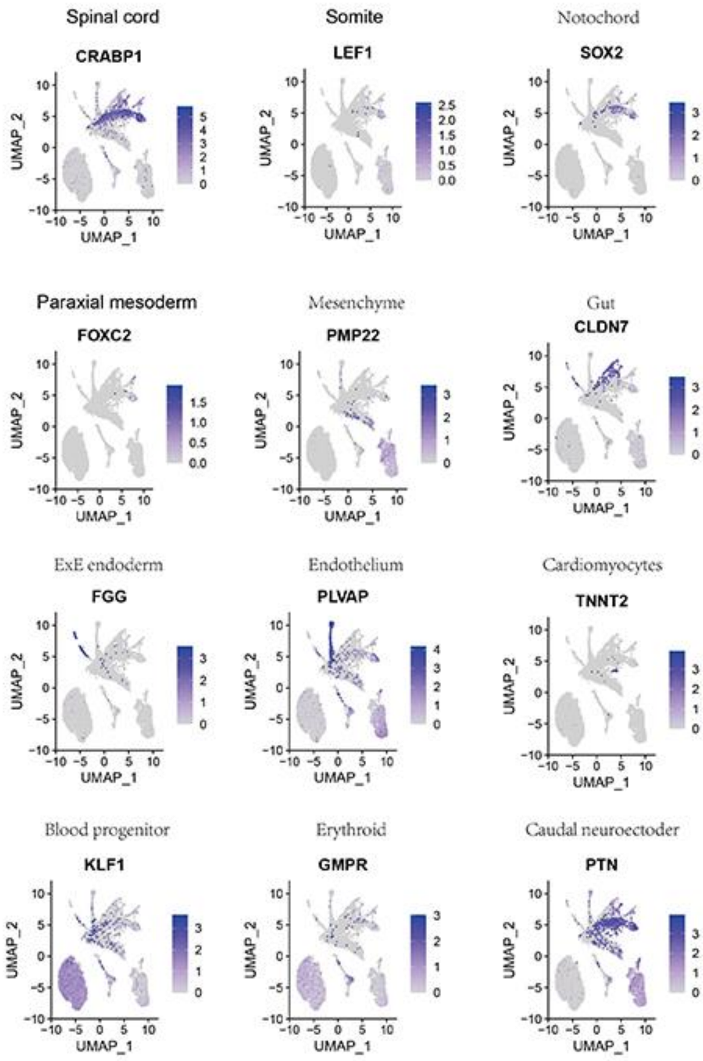

Fig. S2 Cell cluster biomaekers identification analysis

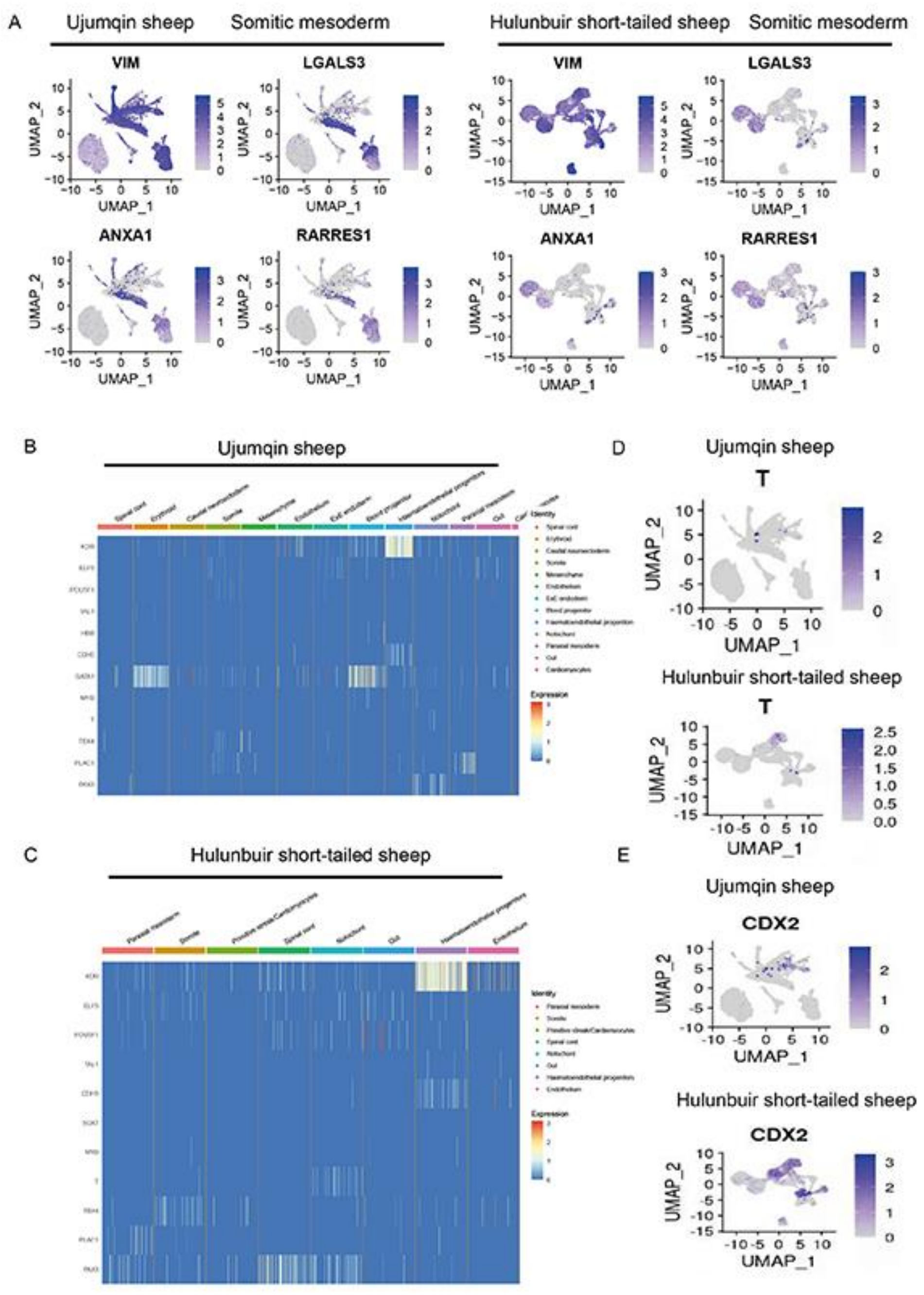

Fig. S3 Related genes expression analysis

(A) Higher expression genes analysis in ujumqin sheep. **(B and C)** PAX3 and T (Brachyury) expression genes analysis in Ujumqin and Hulunbuir sheep. **(D)** T gene expression detection by UMAP. **(E)** CDX2 gene expression detection by UMAP.

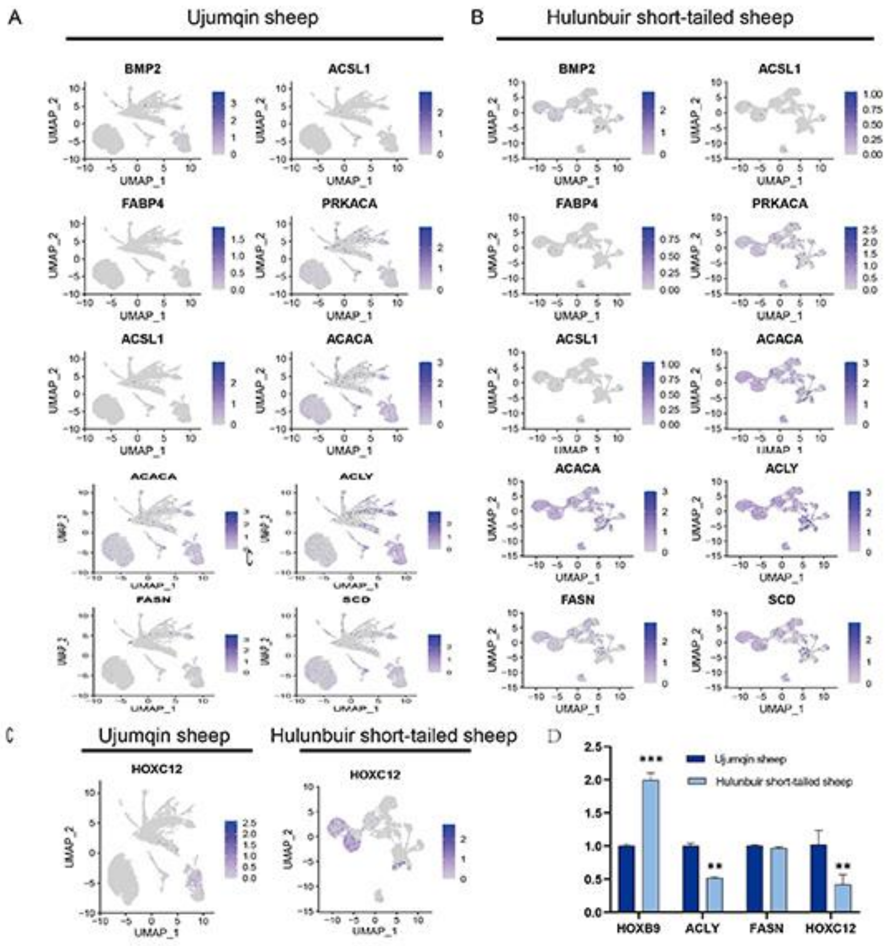

Fig. S4 Hippo and TGF-β related genes expression analysis

(A)YAP1 ,TAZ,ACTB,MYC and AFP expression detection by qPCR. **(B)** HOXB9, ACLY,FASN and HOCX12 expression detection by qPCR. **(C)** BMP2,ACSL1,FABP4,PRKACA,ACACA and ACSL1 expression detection by UMAP in Ujumqin and Hulunuir sheep. **(D)** HOXC12 and TNNC1 expression detection by UMAP in Ujumqin and Hulunuir sheep.

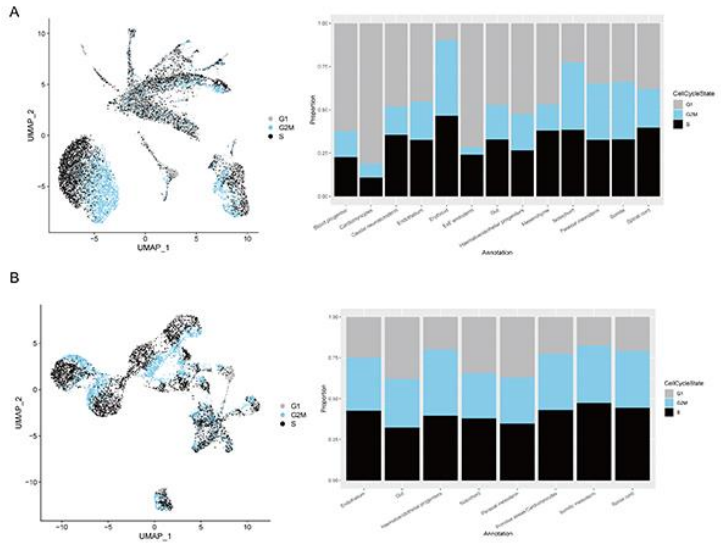

Fig. S5 Cell cycle metrics on Ujumqin and Hulunuir sheep cells.

(A) Cell cycle states in Ujumqin sheep cells projected on UMAP. **(B)** Cell cycle states in Hulunbuir sheep cells projected on UMAP.
